# Intranasal CBT101 demonstrates favorable safety and neurophysiological evidence of central activity in healthy male volunteers

**DOI:** 10.3389/fneur.2026.1940627

**Published:** 2026-10-22

**Authors:** Henri Benech, Lila Krebs-Drouot, Geoffrey Viardot, Valérie Bertaina-Anglade, Clara Lhotellier, Victoria Flament, Camille Roucairol, Aloïse Mabondzo, Jean-Louis Pinquier, Thomas Joudinaud

**Affiliations:** 1 Ceres-Brain Therapeutics, ICM, Hôpital Pitié-Salpêtrière, Paris, France; 2 Eurofins Optimed, Gières, France; 3 Biotrial Neuroscience, Didenheim, France; 4 Biotrial Neuroscience, Rennes, France; 5 Université Paris-Saclay, CEA, INRAE, Département Médicaments et Technologies pour la Santé, SPI/LENIT, Gif-sur-Yvette, France; 6 Decisions R&D consulting, Ile aux Moines, France

**Keywords:** CBT101, cerebral activity, qEEG, safety, phase 1

## Abstract

**Objectives:**

CBT101 is a creatine prodrug under development for disorders driven by impaired neuronal energy homeostasis, with initial development focused on creatine transporter deficiency and amyotrophic lateral sclerosis. This first-in-human study aimed to assess the safety and tolerability of single and repeated ascending intranasal doses of CBT101 in healthy male volunteers and explored neurophysiological evidence of central pharmacodynamic activity using quantitative EEG (qEEG) and event-related potentials (ERPs).

**Methods:**

This was a randomized, double-blind, single-center, placebo-controlled phase 1 trial including single ascending dose (SAD) and multiple ascending dose (MAD) parts. Forty-eight healthy male volunteers were randomized 3:1 to CBT101 or placebo. CBT101 was administered intranasally at 4.2, 8.4, or 12.6 mg in the SAD part and at 4.2, 8.4, or 12.6 mg/day for 14 days in the MAD part. The primary objective was safety and tolerability. Secondary analyses assessed quantitative EEG (qEEG) and event-related potentials (ERPs) as pharmacodynamic evaluations.

**Results:**

No serious or severe treatment-emergent adverse events occurred, no dose-limiting toxicity was observed, and no clinically relevant safety findings were reported. As expected, plasma CBT101 concentrations were below the limit of quantification in all analyzed samples. qEEG analyses identified an acute post-dose increase in relative beta activity after single dosing. After repeated dosing, the qEEG profile evolved toward a more sustained pattern characterized by beta-1 modulation and early gamma-band changes. ERP/P300 analyses showed a transient decrease in P300 latency, with no statistically significant change in P300 amplitude.

**Conclusion:**

Intranasal CBT101 was safe and well tolerated across the evaluated SAD and MAD dose ranges in healthy male volunteers. Convergent qEEG and ERP findings provided early neurophysiological evidence of central pharmacodynamic engagement. These findings support continued clinical development of CBT101 for disorders associated with impaired neuronal energy homeostasis.

**Clinical trial registration:**

ClinicalTrials.gov, identifier: NCT07050316.

## 1 Introduction

Impaired neuronal energy homeostasis is a central feature of several disorders affecting the central nervous system (CNS), including creatine transporter deficiency (CTD) and amyotrophic lateral sclerosis (ALS). In these diseases, impaired intracellular energy buffering contributes to neuronal dysfunction and vulnerability. Given the central role of creatine in the phosphocreatine-creatine shuttle, the main system supporting ATP regeneration, strategies aimed at improving its brain availability remain of considerable therapeutic interest.

Creatine is a central component of cellular energy homeostasis (1). Synthesized mainly from arginine, glycine, and methionine, it is phosphorylated to phosphocreatine, which serves as a rapidly mobilizable energy buffer for ATP regeneration and supports the transfer of high-energy phosphates from mitochondria to sites of cellular energy use. Beyond this central bioenergetic role, creatine has also been implicated in neuroprotection across several CNS disorders, such as neurodegenerative diseases, traumatic brain injury, stroke, and age-related or other cognitive impairments (1, 2). Cellular creatine uptake depends on the creatine transporter (3), which is essential in tissues with limited endogenous synthesis such as the brain (4). This transporter may become saturated under physiological conditions (5) and is directly impaired in CTD, thereby limiting the ability of conventional creatine supplementation to achieve effective cerebral delivery in healthy subjects (6). Mitochondrial dysfunction and/or impaired brain creatine availability has also been reported in several brain disorders (7), and neurologic and neurodegenerative diseases, such as Huntington disease (8), ALS (9), Parkinson’s disease (10) and Alzheimer disease (11).

However, oral creatine supplementation has shown limited utility in CNS applications due to a poor neuronal distribution (12, 13). Effective creatine-based therapies must achieve sufficient brain exposure, reach the relevant target cells, and reproduce the physiological role of endogenous creatine while maintaining an acceptable safety profile. CBT101 is developed to address these challenges (14). Its active pharmaceutical ingredient, dodecyl creatine ester (DCE), is a creatine prodrug (see Figure 1) designed to facilitate creatine neuronal delivery after intranasal administration. This route allows part of the creatine prodrug to be transported along the olfactory and trigeminal nerves, thereby enabling direct access to the brain cells and subsequent release of creatine (15, 16). This mechanism is commonly referred to as the nose-to-brain pathway (17). This combined approach is supported by preclinical studies showing (1) brain delivery of DCE and/or creatine in non-human primates and particularly in their cortical neurons (15) and (2) pharmacological activity in preclinical SLC6A8-KO mouse models of CTD (13, 15), as well as in the 6-OHDA-Parkinson’s rat model (18). Given the high chemical instability of this prodrug, systemic exposure to CBT101 was not expected to reliably reflect CNS penetration. Exploratory neurophysiological measures were therefore incorporated to provide an early assessment of central pharmacodynamic engagement in humans. Exploratory pharmacodynamic assessments were quantitative EEG (qEEG), and event-related potentials (ERPs), with particular focus on the P300 component. Here, we report the first-in-human evaluation of intranasal CBT101 in healthy male volunteers in the setting of a phase 1 clinical study. This study was intended to assess systemic and local safety as well as tolerability after single and repeated ascending doses, and also to characterize early central pharmacodynamic effects of this CNS-directed CBT101 approach in humans. Furthermore, it established an early clinical translational framework integrating safety findings and neurophysiological evidence of central activity, thereby supporting further development in disorders associated with impaired neuronal energy homeostasis.

**Figure 1:**
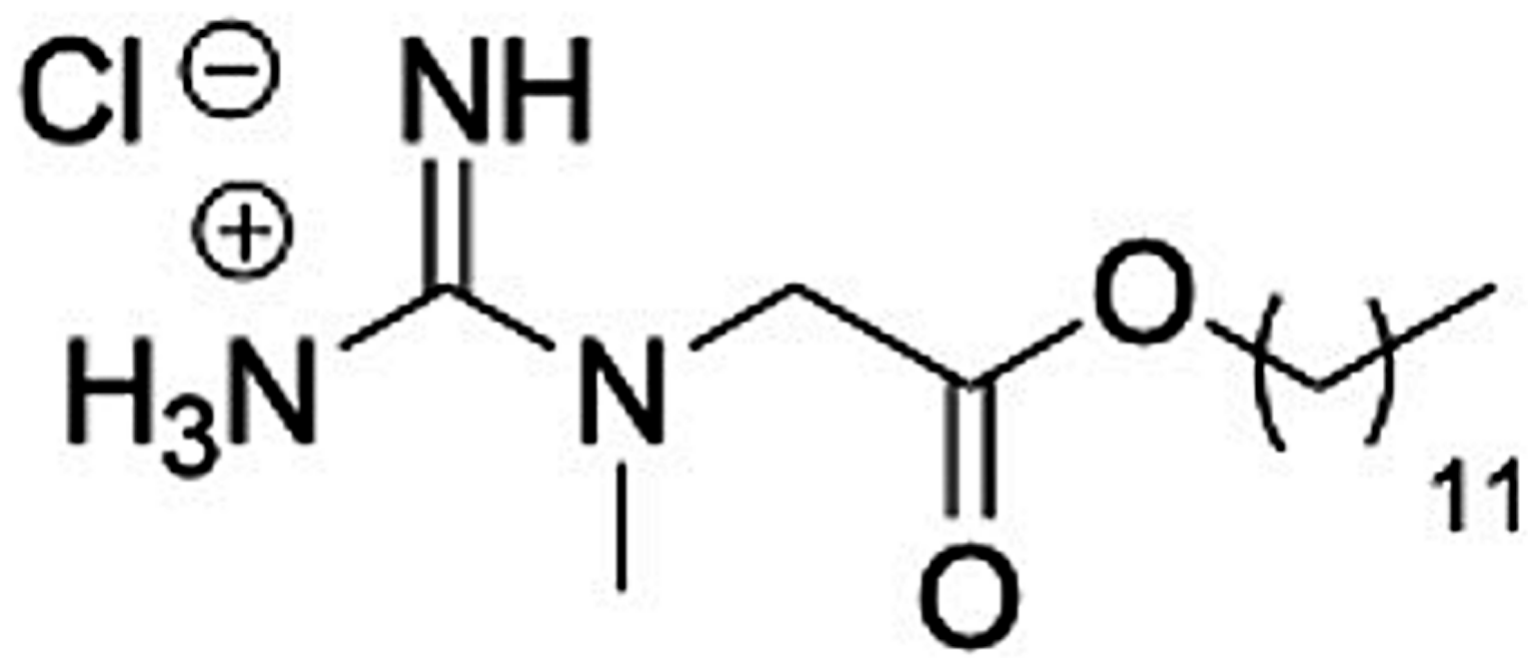
Chemical formula of dodecyl creatine ester, the active principle of CBT101.

## 2 Materials and methods

### 2.1 Study approval

This randomized, double-blind, placebo-controlled, single-center phase 1 study of intranasal CBT101 in healthy male volunteers (EUCT No. 2024-515549-40-00; ClinicalTrials.gov identifier: NCT07050316) was conducted at Eurofins Optimed, Gières, France, in 2025. The study was conducted in accordance with Good Clinical Practice (GCP), the Declaration of Helsinki, and applicable European regulatory requirements. The protocol was approved by the South-West and Over-Sea IV French ethics committee, and all participants provided written informed consent prior to inclusion in the study.

### 2.2 Trial design

The trial included two sequential parts: a single ascending dose (SAD) part and a multiple ascending dose (MAD) part. Each part enrolled 24 subjects, with 18 randomized to CBT101 and 6 to placebo in a 3:1 ratio across three sequential cohorts of 8 subjects each. In part 1 (SAD), subjects received a single administration on Day 1. In part 2 (MAD), subjects received repeated dosing from Day 1 to Day 14, with follow-up through Day 23. Expected individual participation duration was approximately 4 weeks for the SAD cohort and 6 weeks for the MAD cohort.

### 2.3 Trial participants

Eligible participants were healthy male volunteers aged 18 to 55 years, with body weight between 50 and 95 kg and body mass index (BMI) 18.0 to 30.0 kg/m^2^. Participants were required to be judged healthy on medical history, physical examination, vital signs, ECG, laboratory testing, and nasal examination. Serum creatinine had to be within the normal range at screening. Key exclusion criteria included clinically relevant medical history or abnormal findings at screening, clinically significant nasal abnormalities, use of creatine-containing dietary supplements during the study period, and any condition judged by the investigator to interfere with study participation or interpretation of safety data. In the eligible population (*N* = 48), mean age was 36.7 years (SD 10.1), mean body weight 77.9 kg (SD 8.4), mean height 178.6 cm (SD 6.9), and mean BMI 24.4 kg/m^2^ (SD 2.2). All participants were male.

This study was conducted in healthy male volunteers. The inclusion of only male participants was chosen to reduce biological variability in this early phase 1 study focused on safety and exploratory pharmacodynamic assessments. The decision was not based on an expected sex-specific mechanism of action. CBT101 is designed to deliver creatine, an endogenous compound involved in a ubiquitous bioenergetic pathway rather than a sex-specific mechanism, the findings may be relevant beyond males. In addition, nonclinical toxicology studies did not identify a sex-specific safety concern relevant to clinical dose selection. Future studies will include both male and female participants at the same dosage level and will use the tolerated clinical dose range identified in the present study, with appropriate safety monitoring.

### 2.4 Trial drug

CBT101 consists of the dodecyl creatine ester hydrochloride (14) formulated as a particulate oil-in-water emulsion (15). The drug product was filled into CPS pumps (Aptar, France) constituting an integral drug-device combination product. Identity and purity testing was performed on both the drug substance and the drug product in accordance with predefined specifications compliant with applicable ICH recommendations.

CBT101 nasal spray was administered intranasally by an investigator. Each puff delivered 70 μL of nasal formulation, corresponding to 2.1 mg CBT101. In SAD, cohort 1 received 1 puff in each nostril (total 4.2 mg), cohort 2 received 2 puffs in each nostril (total 8.4 mg), and cohort 3 received 3 puffs in each nostril (total 12.6 mg). In MAD, cohort 4 received 1 puff in each nostril once daily (4.2 mg/day), cohort 5 received 1 puff in each nostril twice daily (8.4 mg/day), and cohort 6 received 1 puff in each nostril three times daily (12.6 mg/day). Placebo was administered with matching schedules. In the MAD part, the BID and TID schedules were selected to increase the total daily dose while maintaining an acceptable volume per intranasal administration and limiting the risk of local overflow or posterior drainage. These schedules were not based on systemic parent-compound half-life, as plasma exposure to the parent compound was not expected to reliably reflect CNS delivery for this chemically labile intranasal prodrug.

Administration was performed in the seated position under supervision. Morning dosing took place under fasting conditions around 8:00 a.m., followed 10 min later by breakfast. In SAD cohorts 2 and 3, a 5-min interval was required between pump series. In MAD, evening dosing occurred around 8:00 p.m. For the highest daily dose cohort, an additional midday administration was performed around noon.

### 2.5 Safety assessments

Safety and tolerability were evaluated by monitoring TEAEs, clinical laboratory tests, vital signs, ECGs, physical examinations, body weight and local nasal tolerability, including rhinoscopy. More precisely, local nasal tolerability was assessed by nasal examination, including rhinoscopy, at screening/baseline and at protocol-defined post-dose time points. Nasal findings were evaluated by comparison with baseline, with particular attention to mucosal inflammation, congestion, irritation, discharge, bleeding, or other local abnormalities. Clinically relevant post-treatment changes were recorded as adverse events when appropriate. Predefined dose-escalation stopping criteria were included in the protocol. More precisely, dose escalation was governed by predefined safety stopping criteria. Escalation to the next dose level could be withheld or stopped in the event of clinically significant adverse events, severe or serious adverse events considered related to study treatment, clinically relevant ECG, vital sign or laboratory abnormalities, or clinically significant local nasal tolerability findings.

### 2.6 Bioanalysis

In the SAD part, blood samples for bioanalysis and pharmacokinetic analysis were collected pre-dose and at 5 min, 30 min, 1 h, 2 h, 3 h, 4 h, 8 h, 12 h, and 24 h post-dose. In the MAD part, blood samples were collected on Day 14 at pre-dose and at the same post-dose time points up to 24 h. Plasma concentrations of CBT101 were measured in acidified plasma using an LC–MS/MS assay validated in accordance with ICH M10 guidelines, with a lower limit of quantification of 1 ng/mL. These analyses were performed at the Oncodesign Services center (Villebon-Sur-Yvette, France). Creatine and creatinine were also exploratorily assayed at the Ceres bioanalytical facility in a subset of plasma samples as previously described (15).

### 2.7 Pharmacodynamic assessments

Exploratory pharmacodynamic assessments included quantitative electroencephalography (qEEG) in both the SAD and MAD parts and event-related potentials (ERP/P300) in the MAD part. In the SAD part, qEEG was performed on Day 1 pre-dose, 1 and 3 h post-dose. In the MAD part, qEEG was performed on Day 1 pre-dose, on Day 2 and Day 13 at 1 and 3 h post-dose. For the auditory oddball paradigm, P300 EEG recordings were performed Day 2 and Day 13 of the MAD part, at 1 and 3 h post-dose. EEG recording and processing: During data acquisition, subjects were seated on an armchair bed in a supine or semi-supine position, in a quiet environment, and instructed to remain calm, quiet, relaxed, and awake whilst refraining from head movement, excessive eye blinks and any other unnecessary muscular activity. Resting EEG was acquired for 10 min during two consecutive 5-min eyes-closed and eyes-open sessions. EEG signals were collected using EEG caps including 28 EEG electrodes, following the standard international 10–20 system (Grael® EEG 4 K systems from Compumedics; pass band: 0.1–70 Hz; sampling rate: 512 Hz) plus nine additional electrodes. All these electrodes were used to generate brain maps for spatial visualization of treatment effect, while more in-depth analyses focused on Fz, Cz, Pz and a predefined central area comprising 13 electrodes (F3, Fz, F4, FC1, FC2, C3, Cz, C4, CP1, CP2, P3, Pz and P4). Impedances of all electrodes were kept lower than 20 kΩ. In order to improve artifact rejection, two electro-oculographic channels were simultaneously recorded to monitor ocular movements and eye blinks. In order to reduce electromagnetic noise due to the main power supply, a digital narrow 50 Hz-centered notch filter was applied to data. EEG data were first preprocessed by performing filtering, epoching (data split in 2 s epochs), and various steps of manual (visual inspection and rejection) and automatic artifact reduction and rejection. All these steps of artifact rejection and ERP constructions were carried out using MATLAB. Next, the clean EEG signals were used to estimate the average spectra and to estimate absolute and relative powers on each band of interest [frequency bands defined according to IPEG guideline for qEEG from 0.5 to 100 Hz (19)]. Spectral analyses covered absolute and relative power in delta, theta, alpha, beta, beta-1, beta-2, beta-3, gamma, high-gamma and total bands, as well as ASI [alpha/(delta+theta)], TBR (theta/beta ratio) and TGR (theta/gamma ratio) parameters. To assess the P300 wave ERP, the subject had to perform an auditory oddball paradigm with standard tones of 500 Hz and oddball/target tones of 2000 Hz, each 100 ms in duration. The sound level was 85 dB SPL with 5 ms rise and fall times. The inter-stimulus interval was randomized between 1,200 and 1,900 ms. Oddball made up 15% of presentations. ERP P300 latency (ms) and amplitude (μV) were estimated by extracting the time point corresponding to the largest positive peak within the time interval of 250 ms and 480 ms. Steps of EEG processing were the same except that EEG was segmented in 1.5 s epochs, artifact rejection was primarily done manually, and a baseline correction was performed by subtracting the average amplitude value of the prior stimulus period of [−100; 0 ms]. Epochs were then grouped based on their corresponding stimuli type (i.e., standard or oddball). The P300 ERP was characterized by its amplitude and latency. For the auditory oddball paradigm, ERP/P300 recordings were performed at D1 pre-dose baseline and during the MAD part at Day 2 and Day 13, 1 h and 3 h post-dose.

### 2.8 Statistical analysis

All statistical analyses were performed using SAS software version 9.4 (SAS institute Inc., Cary, NC, United States). This phase 1 study was exploratory and no formal hypothesis-driven sample size calculation was performed. Safety analyses were descriptive. Parent-compound pharmacokinetic parameters were planned to be derived when plasma concentrations permitted reliable estimation. For each part of the study, the EEG analyses to assess the effects of CBT101 were performed with a mixed model for repeated measures on log-transformed data with timepoint, treatment*timepoint interaction and baseline value as fixed effects, and participant as random effect. The model was run for each electrode, each condition (eyes closed and eyes open) and each qEEG and ERP parameter. For each electrode, each parameter and each condition, ratios of adjusted means of each CBT101 dose over placebo at each timepoint were derived (after back transformation when applicable, corresponding to the difference in the log-scale) with corresponding *p*-values. No adjustment of *p*-values was used. Then brain maps were generated representing the result of these comparisons per electrode and condition at each timepoint. As described above, data presented in the results section are placebo-difference brain maps of pooled treatment time-course summaries. A post-hoc pooled-dose analysis was conducted to increase sensitivity for detecting treatment-related changes in EEG signals.

## 3 Results

### 3.1 Subject disposition and baseline characteristics

Baseline demographic characteristics are summarized in Table 1. A total of 98 subjects were screened and 48 were randomized. In the SAD part, 50 subjects were screened and 24 randomized (18 CBT101, 6 placebo); all completed the study. In the MAD part, 46 subjects were screened and 24 randomized (18 CBT101, 6 placebo); 23 completed the study through Day 23. One subject in cohort 5 (8.4 mg/day) withdrew on Day 8 after discontinuation of treatment on Day 4 because of an adverse event judged unlikely related to study treatment. No major protocol deviations occurred. Participants were representative of a healthy male volunteer population, with normal or non-clinically significant baseline findings on physical examination, laboratory testing, vital signs, ECG, and nasal examination.

**Table 1:** Baseline demographics and clinical characteristics of study participants.

Characteristic	Total (N = 48)
Age, years, mean (SD)	36.7 (10.1)
Age range, years	21–54
Body weight, kg, mean (SD)	77.9 (8.4)
Height, cm, mean (SD)	178.6 (6.9)
Body mass index, kg/m^2^, mean (SD)	24.4 (2.2)
Male sex, *n* (%)	48 (100)

### 3.2 Drug administration and exposure

All subjects in the SAD part completed their planned single-dose regimens. In MAD, all subjects in cohorts 4 and 6 completed the full 14-day schedule; in cohort 5, one CBT101-treated subject discontinued early after a total of 5 administrations. All subjects had a dedicated nasal spray pump, administration was well accepted by participants, and no handling difficulties were reported. No technical problems related to study drug administration, particularly concerning pump failure, clogging, or leakage, were reported. In addition, a post-use usability and acceptability assessment of the CBT101 nasal spray device was conducted. The device was consistently rated as ergonomic, easy to use, and intuitive to handle, and immediate post-administration sensations were reported as favorable, particularly with respect to spray pressure, perceived delivered volume, and product temperature. Overall, these findings support a favorable usability and acceptability profile for the CBT101 nasal spray in its intended clinical setting.

### 3.3 Safety and tolerability

No serious or severe treatment-emergent adverse events (TEAEs) occurred (see Table 2). In the SAD part, 4 TEAEs were reported in 3/24 subjects: one placebo-treated subject and two CBT101-treated subjects. All SAD TEAEs were mild and resolved. In the MAD part, 9 TEAEs were reported in 7/24 subjects: two placebo-treated subjects and five CBT101-treated subjects. Eleven events were mild and two were moderate. All resolved without sequelae, and no serious adverse events or dose-limiting toxicities occurred. During the blinded assessment, 10 TEAEs were considered possibly or probably related to study treatment. Two occurred under placebo, namely hyposmia and sneezing. The CBT101-related profile therefore comprised eight events in five participants, consisting of nasal congestion (3 events), nasal inflammation (2), sneezing (1), nasal mucosal erythema (1), and nose tingling reported as paraesthesia (1). Most CBT101-related events resolved within 2 days, although two nasal congestion events persisted for approximately 15 and 19 days (in the highest-dose MAD cohort). All resolved without treatment, sequelae, nor reported functional consequences. All resolved without corrective treatment or additional measures and had no reported functional consequences. None led to dose reduction, treatment interruption, or study withdrawal. One nasal congestion event was moderate; all other CBT101-related events were mild. One CBT101-treated subject in MAD cohort 5 discontinued treatment because of symptomatic orthostatic hypotension, which was finally judged unlikely related to treatment. Overall, the TEAE pattern was dominated by transient nasal/upper respiratory events, with no dose-limiting toxicity and no evidence of increased TEAE frequency with CBT101 versus placebo. Physical examination showed no clinically relevant abnormalities. ECG findings were not considered clinically relevant. Vital sign abnormalities were generally without clinical relevance, except for the single case of orthostatic hypotension event described above. Nasal examinations showed a small number of clinically relevant changes linked to transient local TEAEs, including bilateral nostril inflammation after paresthesia in one SAD subject and inflammation in one MAD subject. Overall, intranasal CBT101 was considered safe and well tolerated across all tested dose levels, with no dose-limiting toxicity observed. The highest tested doses, 12.6 mg as a single dose and 12.6 mg/day for 14 days, were well tolerated, and no dose-limiting toxicity was observed.

**Table 2:** Treatment-emergent adverse events by study part. Values are *n* (%) [number of events].

Event category/SOC/preferred term	Placebo *N* = 6	CBT101 4.2 mg *N* = 6	CBT101 8.4 mg *N* = 6	CBT101 12.6 mg *N* = 6	CBT101 all *N* = 18	Total SAD *N* = 24
At least one TEAE	1 (16.7) [1]	1 (16.7) [1]	1 (16.7) [2]	0	2 (11.1) [3]	3 (12.5) [4]
At least one related TEAE*	1 (16.7) [1]	1 (16.7) [1]	1 (16.7) [2]	0	2 (11.1) [3]	3 (12.5) [4]
At least one mild TEAE	1 (16.7) [1]	1 (16.7) [1]	1 (16.7) [2]	0	2 (11.1) [3]	3 (12.5) [4]
At least one moderate TEAE	0	0	0	0	0	0
At least one severe or serious TEAE	0	0	0	0	0	0
TEAE leading to treatment discontinuation	0	0	0	0	0	0
Respiratory, thoracic, and mediastinal disorders	1 (16.7) [1]	0	1 (16.7) [2]	0	1 (5.6) [2]	2 (8.3) [3]
Nasal congestion	0	0	1 (16.7) [1]	0	1 (5.6) [1]	1 (4.2) [1]
Sneezing	1 (16.7) [1]	0	1 (16.7) [1]	0	1 (5.6) [1]	2 (8.3) [2]
Nervous system disorders	0	1 (16.7) [1]	0	0	1 (5.6) [1]	1 (4.2) [1]
Paresthesia	0	1 (16.7) [1]	0	0	1 (5.6) [1]	1 (4.2) [1]
At least one TEAE	2 (33.3) [2]	1 (16.7) [1]	2 (33.3) [2]	2 (33.3) [4]	5 (27.8) [7]	7 (29.2) [9]
At least one related TEAE*	1 (16.7) [1]	0	1 (16.7) [1]	2 (33.3) [4]	3 (16.7) [5]	4 (16.7) [6]
At least one mild TEAE	2 (33.3) [2]	1 (16.7) [1]	1 (16.7) [1]	2 (33.3) [3]	4 (22.2) [5]	6 (25.0) [7]
At least one moderate TEAE	0	0	1 (16.7) [1]	1 (16.7) [1]	2 (11.1) [2]	2 (8.3) [2]
At least one severe or serious TEAE	0	0	0	0	0	0
TEAE leading to treatment discontinuation	0	0	1 (16.7) [1]	0	1 (5.6) [1]	1 (4.2) [1]
Respiratory, thoracic, and mediastinal disorders	0	0	1 (16.7) [1]	2 (33.3) [4]	3 (16.7) [5]	3 (12.5) [5]
Nasal congestion	0	0	0	2 (33.3) [2]	2 (11.1) [2]	2 (8.3) [2]
Nasal inflammation	0	0	0	2 (33.3) [2]	2 (11.1) [2]	2 (8.3) [2]
Nasal mucosal disorder	0	0	1 (16.7) [1]	0	1 (5.6) [1]	1 (4.2) [1]
Nervous system disorders	1 (16.7) [1]	0	0	0	0	1 (4.2) [1]
Hyposmia	1 (16.7) [1]	0	0	0	0	1 (4.2) [1]
Hepatobiliary disorders	1 (16.7) [1]	0	0	0	0	1 (4.2) [1]
Hyperbilirubinemia	1 (16.7) [1]	0	0	0	0	1 (4.2) [1]
Infections and infestations	0	1 (16.7) [1]	0	0	1 (5.6) [1]	1 (4.2) [1]
Oral candidiasis	0	1 (16.7) [1]	0	0	1 (5.6) [1]	1 (4.2) [1]
Vascular disorders	0	0	1 (16.7) [1]	0	1 (5.6) [1]	1 (4.2) [1]
Orthostatic hypotension	0	0	1 (16.7) [1]	0	1 (5.6) [1]	1 (4.2) [1]

Percentages were calculated using the column denominator. SOC and preferred terms were coded using MedDRA terminology. Panel A. Single ascending dose (SAD). Panel B. Multiple ascending dose (MAD).

^*^Related TEAEs were investigator-assessed as related to the investigational medicinal product. Because the study was blinded, this category includes placebo events. In SAD, related TEAEs comprised placebo sneezing and CBT101-associated paresthesia, nasal congestion, and sneezing. In MAD, related TEAEs comprised placebo hyposmia and CBT101-associated nasal mucosal disorder, nasal congestion, and nasal inflammation. Orthostatic hypotension in MAD cohort 5 led to treatment discontinuation and was finally judged unlikely related to treatment. No severe TEAEs, serious TEAEs, deaths, or dose-limiting toxicities were reported.

### 3.4 Bioanalysis and Pharmacokinetics

Plasma CBT101 concentrations were below the limit of quantification (1 ng/mL) in all analyzed samples. This was expected since CBT101 is an ester and highly unstable in plasma with a t1/2 of metabolism of 3 min. *in vitro* (20). Consequently, no formal pharmacokinetic parameters could be calculated for the parent compound. Plasma creatine and creatinine concentrations were exploratorily measured in a selected subset of plasma samples. No change in plasma creatine or creatinine levels was observed in the analyzed samples (data not shown).

### 3.5 Pharmacodynamics

Exploratory qEEG analyses were initially reviewed by individual dose cohort versus placebo. As no consistent dose–response pattern emerged across the narrow dose range tested, and given the limited sample size within each cohort, active-dose groups were pooled *post hoc* within each study part to improve sensitivity for detecting treatment-related neurophysiological effects, while post-dose time points were retained separately. This approach was considered biologically plausible given preclinical data indicating an early plateau of CNS engagement with intranasal DCE. Importantly, dose-specific analyses did not reveal contradictory trends, supporting the interpretability of the pooled approach.

In the SAD, qEEG was assessed at pre-dose, and 1 h and 3 h post-dose. The clearest effect of the CBT101 acute treatment was a strong significant effect in the total beta [12.5–30 Hz], beta-1 [12.5–18.5 Hz], and beta-3 [21–30 Hz] relative power observed in the eyes closed and resting condition (Figure 2A). Placebo-difference brain maps of the relative power of the beta3 band demonstrated a most prominent effect in the fronto-parietal zone 1 h post-dose, this effect being strongly decreased 3 h post-dose. In addition, no significant effect was detected in the eyes open condition for absolute or relative power.

**Figure 2:**
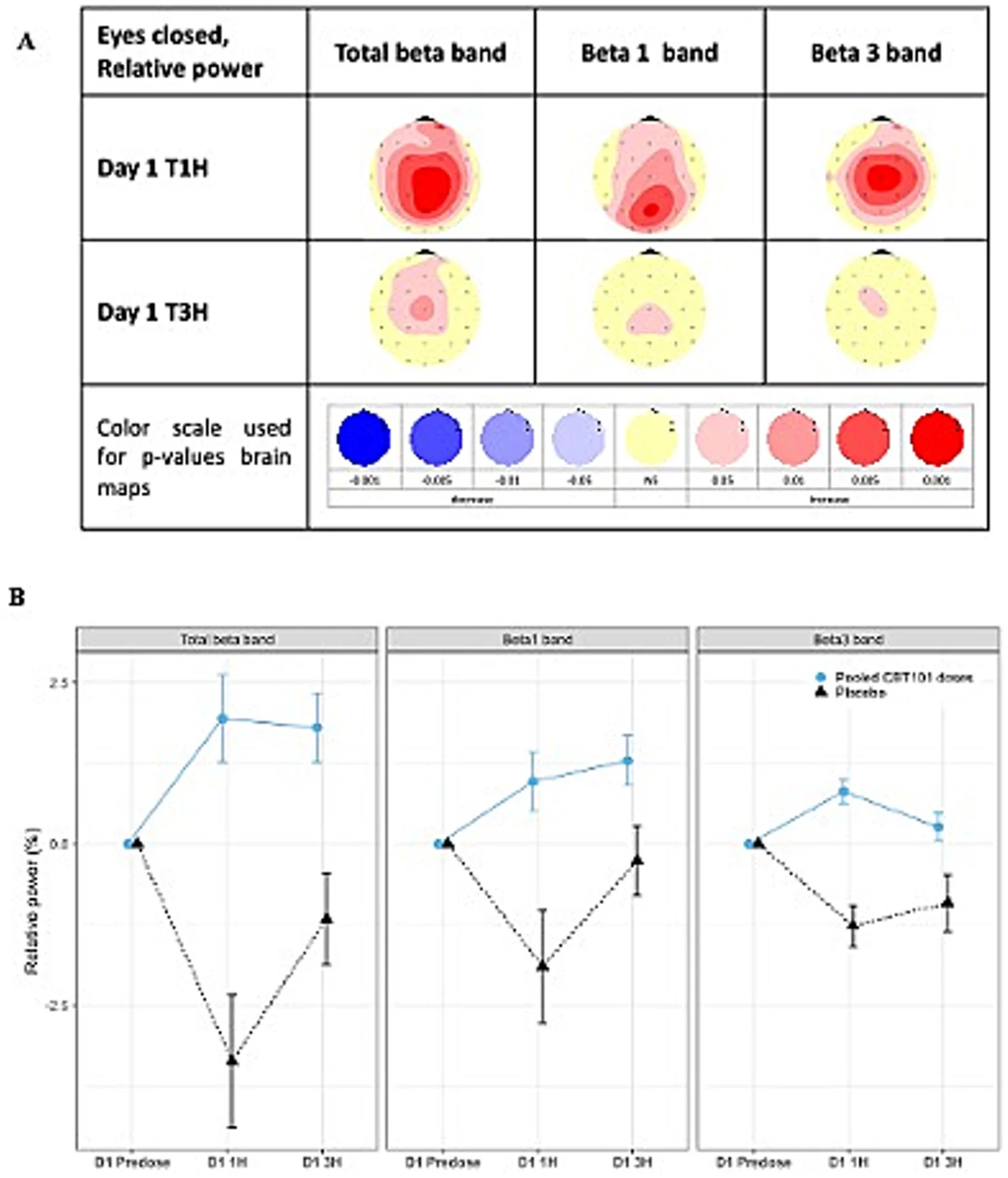
qEEG findings in the SAD part. **(A)** Placebo-difference brain maps showing treatment-related changes in relative power in the total beta, beta-1, and beta-3 bands under resting eyes-closed conditions. **(B)** Corresponding pooled-treatment time courses of relative power in the total beta, beta-1, and beta-3 bands recorded at Cz under eyes-closed conditions at pre-dose, 1 h, and 3 h post-dose and after individual adjustment by predose. Data are mean ± SEM.

The pooled-treatment time courses were directionally consistent with the topographic maps. For example, the time course of the relative beta-1 power on Fz showed clear numerically higher values in the pooled active-treatment group compared to placebo at 1 h post-dose, and a persistent but smaller difference at 3 h post-dose, whereas baseline values were similar in both groups (Figure 2B). Similar directional differences were visible across Cz, Pz, and the central-area pooled-treatment time courses.

Taken together, the SAD data support an EEG post-dose response following a single intranasal administration in the fronto-parietal brain area, resting EEG response being best captured 1 h after administration in the eyes-closed condition, therefore suggesting exploratory evidence of CNS pharmacodynamic activity of CBT101 after single dosing.

Similarly to the SAD part, the main statistically significant effect in the MAD part was observed in relative beta-1 power under eyes-open conditions. This effect was detected mainly in the parietal region at 3 h post-dose on Day 2 (Figure 3A), whereas no clear signal was observed at 1 h post-dose. On the last dosing day (Day 13), a clear difference from placebo was observed in the beta-1 band at both time points, with a more frontotemporal distribution at 1 h post-dose and a more parietal distribution at 3 h post-dose. The corresponding pooled-treatment time courses for the beta-1 band (Figure 3B) confirmed this profile, with relative beta-1 power remaining consistently higher in the CBT101 group than in the placebo group except at baseline, and with the between-group difference being particularly marked at 3 h post-dose on Day 2 while still remaining visible on Day 13.

**Figure 3:**
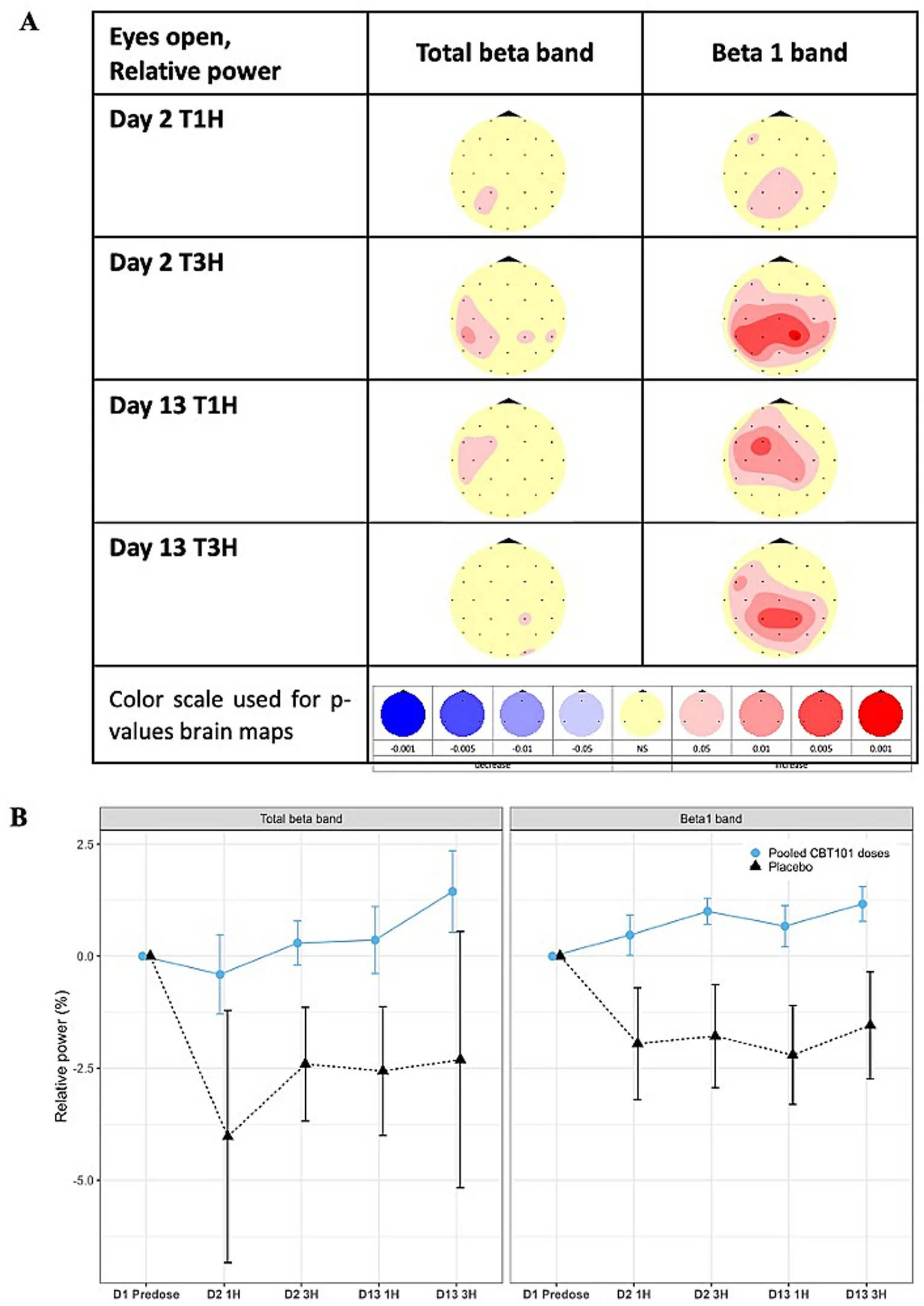
qEEG findings in the MAD part. **(A)** Placebo-difference brain maps showing treatment-related changes in relative power in the total beta and beta-1 bands under eyes-open conditions after multiple intranasal administration of CBT101. **(B)** Corresponding pooled-treatment time courses of relative power in the total beta and beta-1 bands recorded on Cz under eyes-closed conditions on Day 2 and Day 13, 1 h, and 3 h post-dose and after individual adjustment by predose. Data are mean ± SEM.

In addition, some statistically significant differences versus placebo were also observed in relative power of the total gamma [30–40 Hz], high gamma 1 [41–49 Hz] and high gamma 2 [51–80 Hz] bands, for the eyes open condition (Figure 4A). This effect concerned central and temporal brain areas and was mainly observed 3 h post dosing on day 2, as confirmed by the pooled-treatment time courses (Figure 4B).

**Figure 4:**
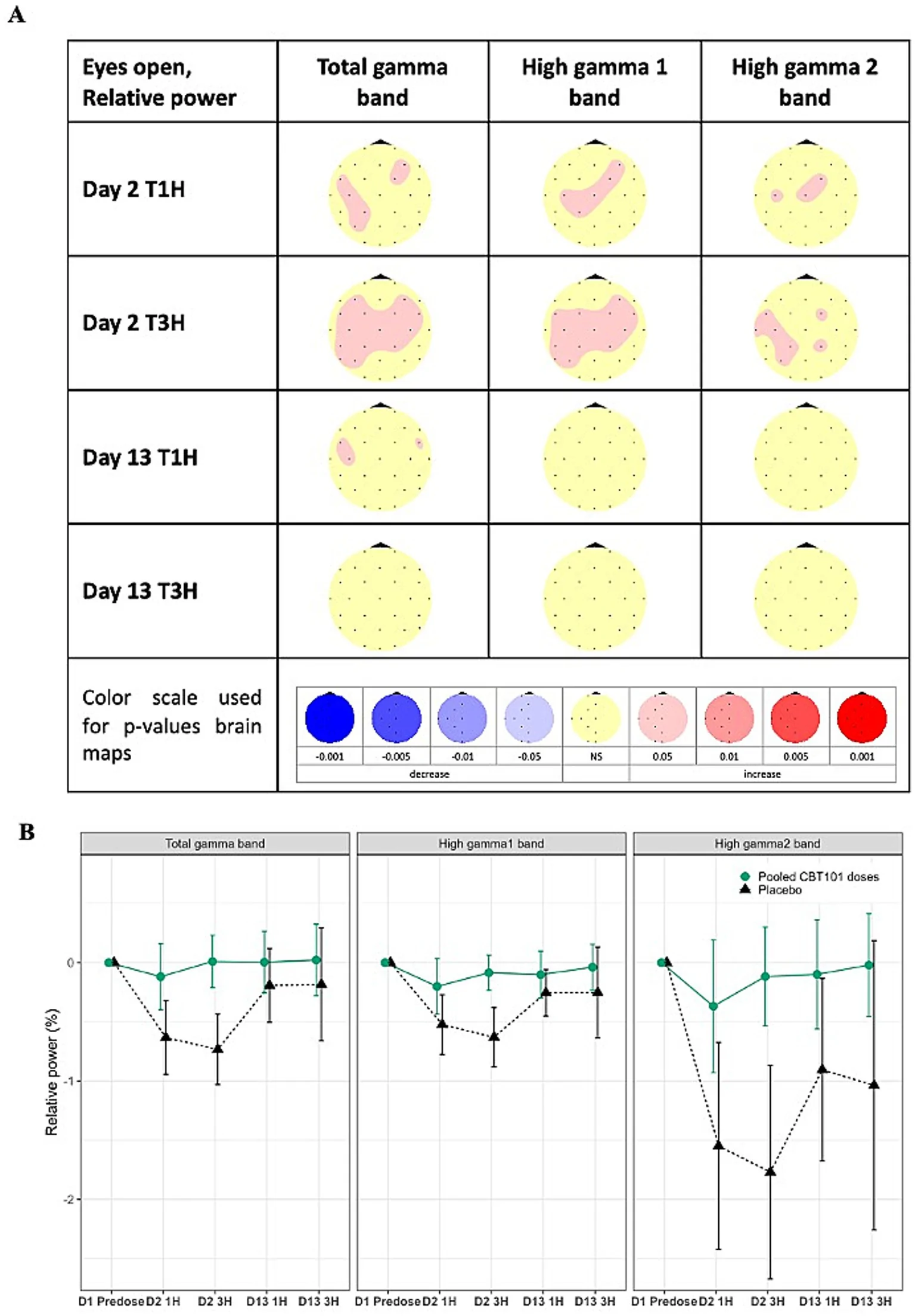
qEEG findings in the MAD part. **(A)** Placebo-difference brain maps showing treatment-related changes in relative power in the total gamma, gamma-1, and gamma-2 bands under eyes-open conditions after multiple intranasal administration of CBT101. **(B)** Corresponding pooled-treatment time courses of relative power in the total gamma, gamma-1 and gamma-2 bands recorded on Cz under eyes-open conditions on Day 2 and Day 13, 1 h, and 3 h post-dose and after individual adjustment by predose. Data are mean ± SEM.

The auditory oddball paradigm was conducted during the MAD part of the study and the corresponding statistical ERP response are presented in the brain maps shown Figure 5. The main finding was a decrease of the P300 latency compared to placebo 1 h post-dosing on day 2 of the chronic treatment. This effect was mainly observed in the fronto-central brain areas and was not maintained over time (lost 2 h later). No statistically significant change in P300 amplitude was detected. Within the overall neurophysiological assessments of the CBT101 effects, ERP/P300 provided complementary exploratory information, whereas the qEEG spectral analyses yielded the most informative treatment-related signals.

**Figure 5:**
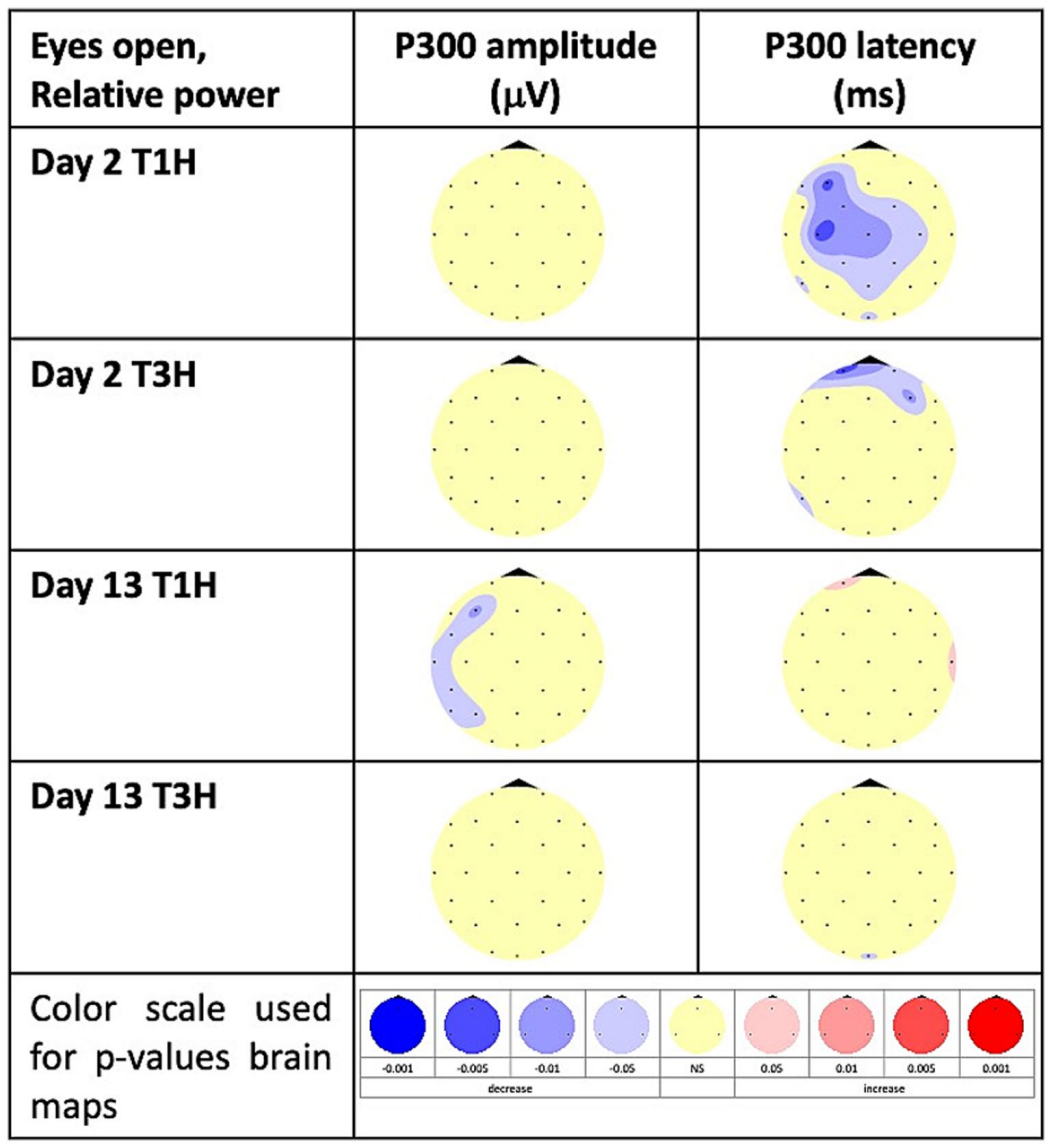
ERP/P300 brain maps in the MAD part. Brain maps show differences versus placebo in baseline-adjusted P300 amplitude (μV) and P300 latency (ms) at Day 2, 1 h post-dose; Day 2, 3 h post-dose; Day 13, 1 h post-dose; and Day 13, 3 h post-dose after repeated intranasal administration of CBT101. Individual post-dose values were adjusted using the corresponding D1 pre-dose ERP/P300 baseline. No pre-dose brain map is shown because the baseline-adjusted pre-dose comparison is centered on zero by construction.

## 4 Discussion

### 4.1 Safety and tolerability

This first-in-human study demonstrated that intranasal CBT101 was safe and well tolerated in healthy male volunteers at single doses up to 12.6 mg administered once and repeated daily doses up to 12.6 mg/day for 14 days. The safety profile was dominated by mild-to-moderate transient local nasal events reported in seven subjects, with no serious adverse events, no clinically relevant ECG findings, and no dose-limiting toxicity. The highest tested doses, 12.6 mg as a single dose and 12.6 mg/day for 14 days, were well tolerated, and no dose-limiting toxicity was observed. The CBT101 nasal spray therefore benefits from a dedicated formulation specifically designed for long-term human use, combining humectants, a very low surfactant concentration, and the absence of preservatives in order to optimize local tolerability (15). As a complement to the qEEG assessments, PEPSY® neurocognitive testing (such as memory, attention, vigilance, balance, oculography (21)) performed at Biotrial facilities did not reveal any detrimental neurocognitive signal or any detectable pharmacodynamic effect on cognitive performance in this healthy volunteer population.

### 4.2 Bioanalysis and Pharmacokinetics

Preclinical studies supported brain-directed delivery (15) and pharmacological activity of CBT101 (16, 22), whereas conventional systemic pharmacokinetics were expected to be only weakly informative because of the known lability of creatine prodrugs (23) and the intended nose-to-brain delivery strategy (22). The absence of quantifiable parent compound in plasma was anticipated. The LC–MS/MS method used for plasma CBT101 measurement has been validated according to ICHM10 requirements with a lower limit of quantification of 1 ng/mL/. However, CBT101 is highly labile in solution (23) and including biological matrices (15) with an *in vitro* t1/2 of metabolism is 3 min (20). Indeed, dodecyl creatine ester (the CBT101’s drug substance) is metabolized by two ways: 1- an hydrolysis of the ester into creatine and dodecanol and 2- a nucleophilic reaction involving the guaninide and the ester function leading to creatinine (the cyclized form of creatine) and dodecanol. Preclinical studies have repeatedly shown that CBT101 reaches the brain with minimal systemic spillover. Therefore, the absence of quantifiable plasma CBT101 drug substance is consistent with the physicochemical properties of DCE and with the intended nose-to-brain delivery strategy, but it limits conventional systemic pharmacokinetic interpretation. Therefore, neurophysiological markers were considered as a more informative approach for demonstrating early human proof of CNS target engagement.

Regarding creatine and creatinine determination. No change in plasma creatine or creatinine levels was observed in the analyzed samples (data not shown). This was not unexpected, given the low doses administered intranasally (4.2 to 12.6 mg/day) and the relatively high endogenous plasma concentrations of creatine (~ 40 μg/mL) and creatinine (~8 μg/mL) in humans (24). For example, even assuming complete systemic absorption of a 12.6 mg intranasal dose, and using the reported apparent volume of distribution of creatine (Vd/F, approximately 18 and 59 L), the expected plasma creatine concentration would be only 0.21 to 0.69 μg/mL, i.e., far below endogenous levels.

### 4.3 Pharmacodynamics

The qEEG findings provide strong evidence of central pharmacodynamic engagement in this study. After single administration, the clearest signal was a transient increase in beta relative power (total beta, beta-1 and beta-3 bands) under the eyes closed condition at 1 h post-dose. In early-phase CNS studies, pharmaco-EEG is a sensitive method for detecting drug-related changes in cortical network activity even in the absence of measurable behavioral effects (25, 26). Traditionally associated with sensorimotor processing, overall beta activity changes (increases/decreases) are increasingly implicated in a wide range of cognitive functions: visual perception, language processing, working memory, long-term memory, decision making, and reward processing (27). This is mechanistically consistent with the expected neurophysiological effects of a creatine prodrug on cortical network function. The modulation of beta and gamma oscillatory activity observed with CBT101 is consistent with the known bioenergetic dependence of fast cortical rhythms. High-frequency oscillations, particularly in the beta–gamma range, rely on metabolically demanding interneuron networks, most notably parvalbumin-positive interneurons, which require rapid ATP regeneration to sustain synchronous firing. Creatine-dependent phosphocreatine buffering plays a key role in supporting these high-energy neuronal processes. Thus, the observed enhancement of beta-1, beta-3, and gamma activity after CBT101 administration aligns with the expected neurophysiological consequences of improved intracellular energy availability. In some of these contexts, beta-band modulations are observed in the low frequency range (beta-1 sub band, 12.5–18.5 Hz), whereas in other cases, changes in beta rhythms are observed at higher frequencies (beta-3 sub band, 21–30 Hz) and behave similarly to gamma activity (>30 Hz), increasing with task-related engagement (28). For example, increased beta power reported in numerous working memory tasks can depend on various task factors (such as memory load) and is also often found in other frequency bands such as the gamma band (29). The fact that this signal was most apparent under the eye closed condition is also compatible with standard resting-state EEG practice, in which oscillatory drug effects are often more readily detected in low-input conditions. Importantly, the bands modulated by CBT101 were not predominantly slow rhythms, but fast beta-range oscillations, which are more directly related to cortical information processing. In this context, modulation of beta-1 and beta-3 activity is consistent with the early effect of a creatine prodrug.

The repeated-dose qEEG profile appeared to extend this early signal rather than simply reproduce the acute pattern observed after first administration. Although placebo-difference topographic maps in the MAD part were less intense and less diffuse than those observed in the SAD part, pooled longitudinal summaries suggested a more sustained activity pattern involving beta-1 (for all chronic dosing duration) and gamma (early in the repeated dosing regimen only) activity, particularly in central brain areas. This temporal evolution is also mechanistically coherent with the expected action of creatine: the emergence of beta-1 and gamma activity after repeated dosing may indicate a more stable and coordinated pattern of neuronal activity under improved energetic support. This interpretation is particularly plausible for gamma oscillations, which are among the most energy-demanding cortical rhythms and are strongly dependent on mitochondrial ATP availability. Overall, the sequence from early total beta/beta-1/beta-3 responses to a repeated-dose beta-1/gamma pattern supports sustained CNS pharmacodynamic activity with continued administration.

Initial dose-by-dose examination of the qEEG dataset did not reveal a consistent gradient of electrophysiological effect across the tested clinical dose range. This absence of a clear dose–response relationship is compatible with the preclinical pharmacology of CBT101. In an SLC6A8-KO mouse model of CTD, increasing the intranasal doses from 0.02 to 0.40 mg per mouse did not produce additional efficacy, suggesting a plateau effect (data not shown). Using the translational framework based on drug amount at the application site (30), this range corresponds to a human equivalent dose of 1.1 to 22.9 mg, encompassing the clinical doses evaluated here (4.2–12.6 mg). Within this first-in-human, safety-driven dose range, the pooling of active doses while retaining separate post-dose time points was therefore considered both biologically and methodologically justified for the main exploratory qEEG analyses.

These findings should be interpreted in the context of a first-in-human phase 1 study. First, the study was not performed in the target patient population and therefore does not address therapeutic relevance directly. In particular, this first-in-human study was conducted exclusively in healthy male volunteers, which limits direct extrapolation to female participants. Nevertheless, CBT101 is designed to deliver creatine, an endogenous guanidino compound involved in cellular energy buffering, and its expected pharmacodynamic activity targets a ubiquitous bioenergetic pathway rather than a sex-specific mechanism. Moreover, conversion of CBT101 into creatine, creatinine, and dodecanol is primarily driven by chemical instability and hydrolysis/cyclization processes, rather than by sex-dependent hepatic metabolic enzymes. The observed adverse events were mainly mild, transient, local nasal, or upper respiratory events, with no systemic safety signal suggesting a mechanism likely to differ substantially between males and females. These considerations support the relevance of the present findings for future development, while potential sex-related differences in tolerability, exposure, or neurophysiological response should be assessed in subsequent patient studies including female participants. Second, the sample size was appropriate for early safety evaluation but was not designed for formal confirmation of pharmacodynamic effects. Third, a limitation is the short duration of repeated dosing. The 14-day MAD part was appropriate for a first-in-human phase 1 assessment of short-term safety, tolerability, and exploratory central pharmacodynamic activity, but it is not sufficient to determine long-term tolerability, sustained pharmacodynamic effects, or therapeutic usefulness in chronic neurological disorders. These points will require longer studies in the intended patient populations.

## 5 Conclusion

Together, these findings establish a coherent early translational profile for CBT101. The compound was safe and well tolerated following both single and repeated intranasal administrations and produced reproducible qEEG signatures consistent with central pharmacodynamic engagement. The convergence between preclinical nose-to-brain delivery data and human neurophysiological effects supports the biological plausibility of this approach. The temporal evolution of the qEEG signature, from an acute beta-1/beta-3 response after single dosing to a more sustained beta-1/gamma pattern after repeated administration, was consistent with adaptive cortical network changes under improved energetic support and with the expected neurophysiological effects of creatine on cognition-relevant brain function. Importantly, the modulation of fast beta- and gamma-range oscillations in healthy volunteers is of particular translational interest for disorders such as CTD and ALS, in which impaired neuronal energy homeostasis is thought to contribute directly to network dysfunction and vulnerability. Collectively, these results support continued clinical development of CBT101 in patient populations with disorders associated with impaired neuronal energy homeostasis.

## Data Availability

The raw data supporting the conclusions of this article will be made available by the authors, without undue reservation.
